# The etiological diagnostic value of metagenomic next-generation sequencing in suspected community-acquired pneumonia

**DOI:** 10.1186/s12879-024-09507-6

**Published:** 2024-06-24

**Authors:** Mengling Liu, Haiyue Zhang, Liangyu Li, Jieyu Mao, Ruiyun Li, Jing Yin, Xiaojun Wu

**Affiliations:** Department of Pulmonary and Critical Care Medicine, Renmin Hospital of Wuhan University, Wuhan, Hubei China

**Keywords:** Metagenomic next-generation sequencing, Bronchoalveolar lavage fluid, Community-acquired pneumonia, Pathogenic identification, Diagnostic value

## Abstract

**Background:**

The emergence of metagenomic next-generation sequencing (mNGS) may provide a promising tool for early and comprehensive identification of the causative pathogen in community-acquired pneumonia (CAP). In this study, we aim to further evaluate the etiological diagnostic value of mNGS in suspected CAP.

**Methods:**

A total of 555 bronchoalveolar lavage fluid (BALF) samples were collected for pathogen detection by mNGS from 541 patients with suspected CAP. The clinical value was assessed based on infection diagnosis and treatment guidance. The diagnostic performance for pathogen identification by mNGS and sputum culture and for tuberculosis (TB) by mNGS and X-pert MTB/RIF were compared. To evaluate the potential for treatment guidance, we analyzed the treatment regimen of patients with suspected CAP, including imaging changes of lung after empirical antibacterial therapy, intensified regimen, antifungal treatment, and a 1-year follow up for patients with unconfirmed diagnosis and non-improvement imaging after anti-infective treatment and patients with high suspicion of TB or NTM infection who were transferred to the Wuhan Pulmonary Hospital for further diagnosis and even anti-mycobacterium therapy.

**Results:**

Of the 516 BALF samples that were analyzed by both mNGS and sputum culture, the positivity rate of mNGS was significantly higher than that of sputum culture (79.1% vs. 11.4%, *P* = 0.001). A total of 48 samples from patients with confirmed TB were analyzed by both mNGS and X-pert MTB/RIF, and the sensitivity of mNGS for the diagnosis of active TB was significantly lower than that of X-pert MTB/RIF (64.6% vs. 85.4%, *P* = 0.031). Of the 106 pathogen-negative cases, 48 were ultimately considered non-infectious diseases, with a negative predictive value of 45.3%. Of the 381 pathogen-positive cases, 311 were eventually diagnosed as CAP, with a positive predictive value of 81.6%. A total of 487 patients were included in the evaluation of the therapeutic effect, and 67.1% improved with initial empirical antibiotic treatment. Of the 163 patients in which bacteria were detected, 77.9% improved with antibacterial therapy; of the 85 patients in which fungi were detected, 12.9% achieved remission after antifungal therapy.

**Conclusions:**

Overall, mNGS had unique advantages in the detection of suspected CAP pathogens. However, mNGS was not superior to X-pert MTB/RIF for the diagnosis of TB. In addition, mNGS was not necessary as a routine test for all patients admitted with suspected CAP. Furthermore, when fungi are detected by mNGS, antifungal therapy should be cautious.

## Background

Pneumonia is one of the leading causes of morbidity and mortality worldwide, and choosing an effective management strategy is complex because of the wide array of potential causative pathogens. Thus, early identification of the causative pathogen is key to early and effective treatment [19]. The identification of pathogens in community-acquired pneumonia (CAP) has traditionally relied on conventional assays such as microbial culture, antigen/antibody assays, and polymerase chain reaction (PCR). However, in current clinical practice, these have limited diagnostic efficiency [8, 21, 26, 32]; therefore, a new diagnostic method with greater efficiency is urgently needed.

Metagenomic next-generation sequencing (mNGS) is a technology for microbial identification that is based on nucleic acid detection, allowing for simultaneous identification of bacteria, fungi, viruses, atypical pathogens [9, 18, 28, 33], parasites, and novel pathogens [24] in clinical specimens. Theoretically, mNGS is capable of hypothesis-free detection of all pathogens in a single assay and has a wider range of detectable pathogens, requires a shorter amount of time, and is less affected by previous antibiotic exposures than other detection methods [5, 17, 29, 30]. Thus, the emergence of mNGS technology provides a promising tool for etiological diagnosis. Although mNGS has been used for the diagnosis of infectious diseases [24], its clinical value for the etiological diagnosis of suspected CAP remains to be explored. Therefore, this study aimed to evaluate the diagnostic value of mNGS for suspected CAP and initiate a preliminary discussion on the related issues.

## Methods

### Study design

This study evaluated 541 patients with suspected CAP who were hospitalized in the Department of Respiratory and Critical Care Medicine at Renmin Hospital of Wuhan University between July and December 2022. The diagnostic criteria for CAP were in accordance with the Chinese guidelines for CAP in adults [10]. The inclusion criteria were as follows: (1) sufficient bronchoalveolar lavage fluid (BALF) collected for laboratory testing within 72 h of admission; (2) informed consent from the patients or their surrogates; and (3) complete clinical information. Patients who could not tolerate electronic bronchoscopy were excluded from this study. This study was approved by the research ethics committee of Renmin Hospital of Wuhan University.

Patients were routinely treated with empirical antibacterial therapy (β-lactam alone or in combination with doxycycline, minocycline, macrolides or respiratory uinolones alone [10]) upon admission, and the treatment effect was evaluated after 5–7 days. The criteria for effective treatment were (1) improvement or absence of clinical symptoms and (2) partial or complete absorption of the lesions on chest imaging. These criteria were evaluated by three respiratory physicians. If initial empirical antibacterial therapy was ineffective, patients were treated with an intensified regimen (carbapenems and other high-grade antibiotics or antibiotic combinations therapy) or antifungal treatment. Of the patients who did not improve with these treatments, puncture biopsy or other relevant tests were performed for definitive diagnosis. Patients who improved after anti-infective treatment, or died, or not improved but have a confirmed diagnosis during hospitalization are not followed up. Patients with unconfirmed diagnosis and non-improvement of imaging after anti-infective treatment during hospitalization and patients with high suspicion of TB or NTM infection who were transferred to the Wuhan Pulmonary Hospital for further diagnosis and even anti-mycobacterium therapy were dynamically observed and followed up for 1 year. Chest imaging changes were the main objective of follow-up, and patients’ recent status were asked at each follow-up visit. Patients received their first imaging tests of lung (CT or DR) 1–3 months after discharge, and then every 3 or 6 months during the subsequent follow-up period. 

### Sample collection and mNGS analysis

BALF samples were obtained during bronchoscopies performed for clinical management, and mNGS was used to detect pathogens in these samples. For patients with a high suspicion of TB based on their clinical symptoms and chest imaging, BALF samples were also sent for X-pert MTB/RIF. Samples for sputum culture were aspirated before obtaining BALF samples under bronchoscopy from patients with sputum.

Briefly, the mNGS procedures were as follows: (1) DNA extraction: DNA was extracted from 0.5 to 3 mL of BALF, according to the instructions of the Sansure DNA Extraction Kit (Sansure Biotech Inc, Changsha, China). (2) Library construction and sequencing: approximately 50 ng of DNA from each sample was used for library construction using the SQK-LSK109 kit according to the manufacturer’s instructions (Oxford Nanopore Technologies, Oxford, UK) and the NEBNext Quick T4 DNA ligase connector enzyme. The 16 S target amplification was approximately 1.5 kb, the internal transcribed spacer (ITS) was approximately 400–800 bp, and the libraries were quantified using Qubit 3.0. Quality control analyses were performed by high-throughput sequencing using the Nanopore MinION sequencer (Oxford Nanopore Technologies, Oxford, UK). (3) Data analysis: sequencing data were split according to sequencing tags, splices were removed, low-quality and duplicate sequences were filtered, and GRCH38 (downloaded from the National Center for Biotechnology Information [NCBI]) pathogen database) was used as the reference genome to remove human host DNA. The pathogen database was the 16 S and ITS gene database (downloaded from NCBI), which included 19,088 bacterial, 8,082 fungal, and 231 non-bacterial pathogen reference genes.

### Statistical analysis

The value of mNGS in patients with suspected CAP was assessed by comparing mNGS results with the patient’s clinical diagnosis and treatment response. Sensitivity was evaluated using 2 × 2 contingency tables, and the McNemar test for discrete variables was applied when appropriate. Data analyses were performed using SPSS (version 26; IBM Corp., Armonk, NY, USA). *P* values < 0.05 were considered statistically significant, and all tests were two-tailed. Figures were constructed with GraphPad Prism (version 9; GraphPad Software, La Jolla, CA, USA) and R (The R Foundation, Vienna, Austria).

## Results

### General patient characteristics

**Fig. 1:**
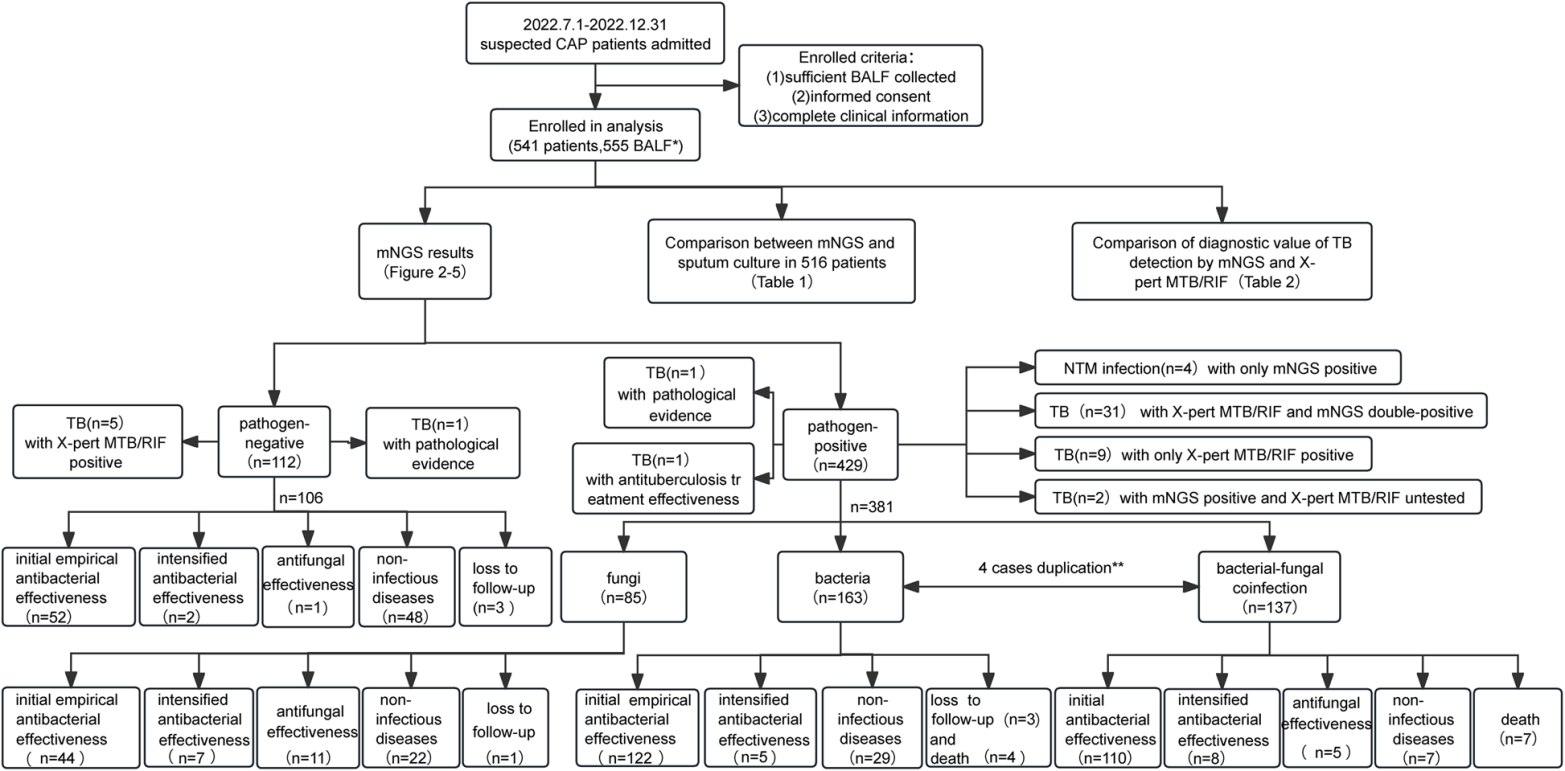
Flow chart of the study design and mNGS results. * Of the 541 patients, 2 BALF samples were collected from 12 patients and 3 samples were collected from 1 patient. ** 4/487 patients had samples sent for mNGS on two separate occasions, with one mNGS assay for bacteria and the other for bacterial-fungal coinfection. Of these 4 patients, 1 responded to initial antibacterial therapy, 2 died, and 1 was diagnosed with a non-infective pulmonary nodule. These 4 patients were double-counted in the bacterial and bacterial-fungal coinfection groups. BALF, bronchoalveolar lavage fluid; CAP, community-acquired pneumonia; mNGS, metagenomic next-generation sequencing; NTM, non-tuberculous mycobacteria; TB, tuberculosis

A total of 541 patients were included in this study, with 271 males and a median age of 58 years (interquartile range: 43–67 years). A total of 555 BALF samples were collected (Fig. 1), with 12 patients having 2 BALF samples and 1 patient with 3, collected based on the patients’ conditions. According to the study design, 93 patients followed up for one year, and 7 patients lost to follow-up.

### Detection of pathogen by mNGS

**Fig. 2:**
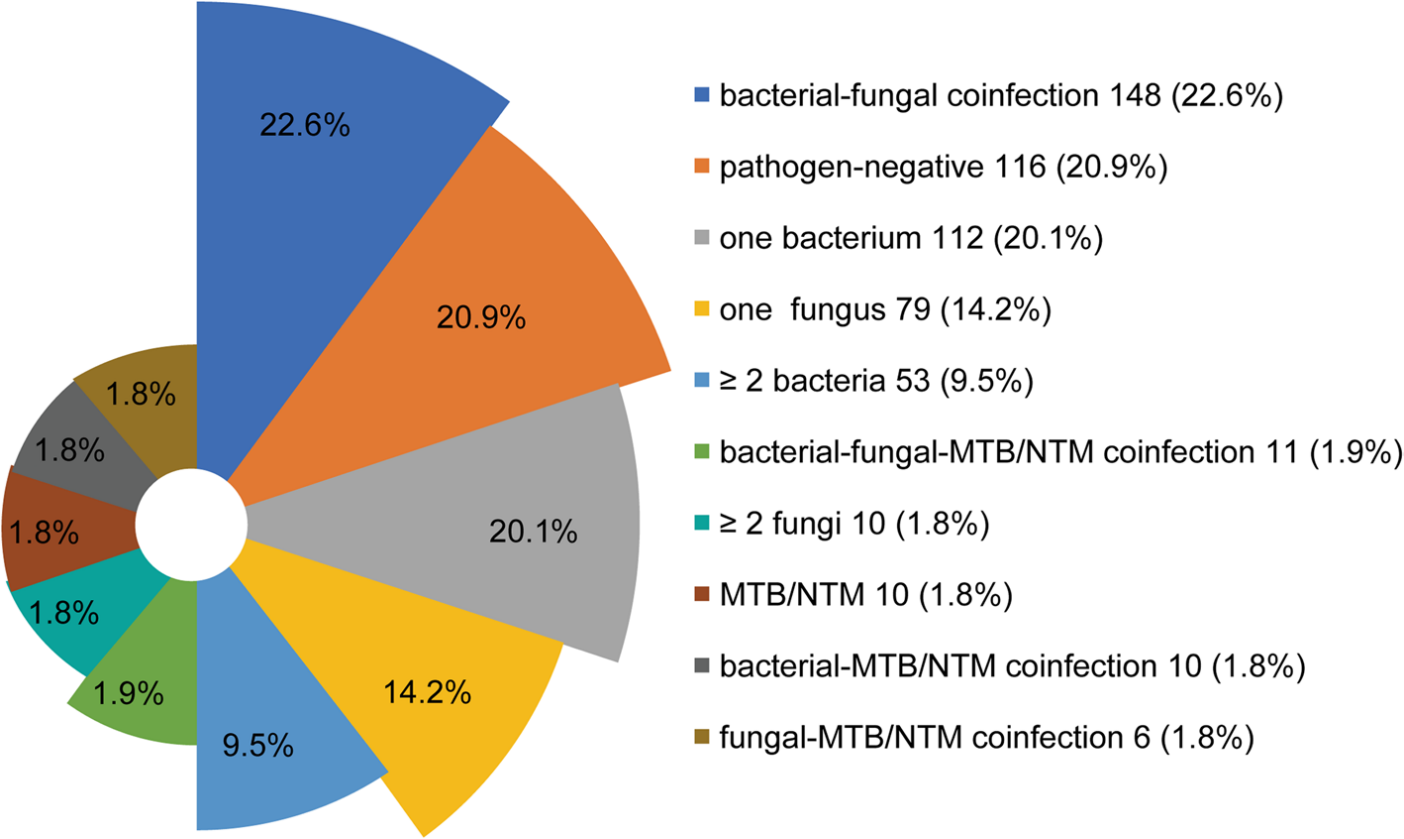
Composition of pathogens detected by mNGS. MTB, Mycobacterium tuberculosis ; NTM, non-tuberculous mycobacteria

**Fig. 3:**
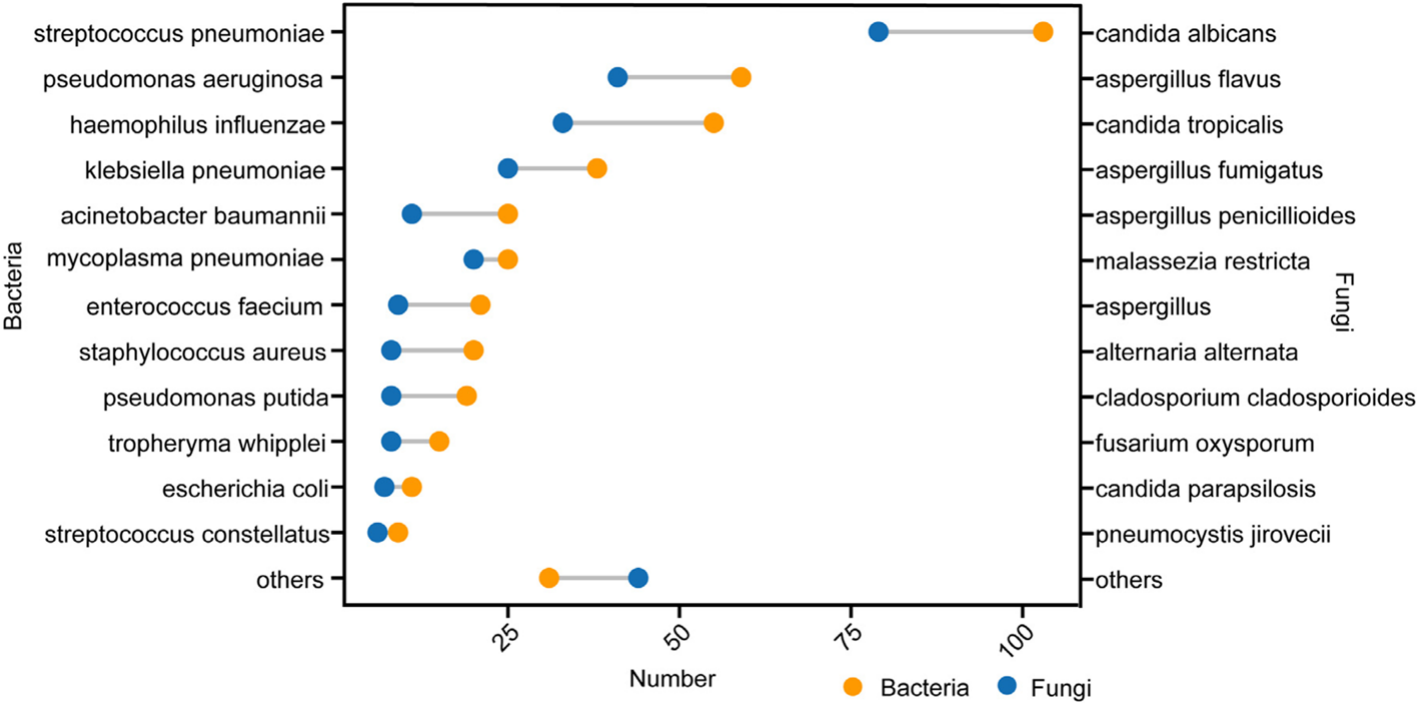
Distribution of bacteria and fungi detected by mNGS

The positivity rate of the 555 BALF samples was 79.1% (*N* = 439), and the negativity rate was 20.9% (*N* = 116). The mNGS results were categorized into bacteria, fungi, and mycobacteria (comprising *Mycobacterium tuberculosis* [MTB] and non-tuberculous mycobacteria [NTM]). Bacteria, fungi, and mycobacteria were detected in 334 (60.2%), 254 (45.8%), and 37 (6.7%) BALF samples, respectively. Only one bacterium was detected in 20.2% (*N* = 112) of the bacteria-positive BALF samples, ≥ 2 bacteria in 9.5% (*N* = 53), and coinfection with other types of pathogens in 30.5% (*N* = 169). Only one fungus was detected in 14.2% (*N* = 79) of the fungi-positive BALF samples, ≥ 2 fungi in 1.8% (*N* = 10), and coinfection with other types of pathogens in 29.7% (*N* = 165). Mycobacterium alone was detected in 1.8% (*N* = 10) of the mycobacterium-positive samples and coinfections with other types of pathogens in 4.9% (*N* = 27). These 37 cases of mycobacterium comprised 33 (5.9%) MTB and 4 (0.7%) NTM (3 intracellular mycobacteria and 1 *Mycobacterium kansasii*). Coinfections were detected in 238 (42.9%) BALF samples, with bacterial-fungal coinfection being the most common (*N* = 148, 26.7%) (Fig. 2). The top three bacteria were *Streptococcus pneumoniae*, *Pseudomonas aeruginosa*, and *Haemophilus influenzae*, and the top three fungi were *Candida albicans*, *Aspergillus flavus*, and *Candida tropicalis* (Fig. 3).

### Pathogen detection: mNGS vs. sputum culture

Of the 541 patients, 25 patients did not have sputum samples for sputum culture. For the remaining patients, pathogens were detected by both mNGS and sputum culture, and mNGS had a significantly higher positivity rate than sputum culture (79.1% vs. 11.4%, respectively, *P* = 0.001). The results of the two methods were consistent in 159 cases (55 double-positive and 104 double-negative), with a concordance accuracy of 30.8% (Table 1). Of the 55 double-positive samples, 72.7% (*N* = 40) showed complete (*N* = 9) or partial (*N* = 31) concordance between mNGS and sputum culture.

**Table 1:** Comparison between mNGS and sputum culture

mNGS	sputum culture		total
	+	-	
+	55	353	408
-	4	104	108
total	59	457	516

*mNGS* metagenomic next-generation sequencing

### Diagnostic value of tuberculosis (TB) detection: mNGS vs. X-pert MTB/RIF

Of the 112 pathogen-negative cases, 1 was pathologically suggestive of TB and 5 were positive by X-pert MTB/RIF. Of the 429 pathogen-positive cases, 1 was pathologically suggestive of TB, 1 improved after empirical anti-tuberculosis therapy, 4 were NTM-positive by mNGS, 31 were double-positive by both mNGS and X-pert MTB/RIF, 9 were only positive by X-pert MTB/RIF, and 2 that were not sent for X-pert MTB/RIF were positive by mNGS. Overall, 50 patients with confirmed or suspected TB and 4 patients with suspected NTM infection were immediately transferred to the Wuhan Pulmonary Hospital for further diagnosis and treatment. Ultimately, after 1 year of follow-up, 50 patients were diagnosed with TB and 4 with NTM infection. Of the 50 TB cases, 48 were tested by both mNGS and X-pert MTB/RIF. The sensitivity for diagnosing TB by mNGS and X-pert MTB/RIF was 64.6% and 85.4%, respectively (*P* = 0.031), and the specificity was 100% for both modalities (Table 2).

**Table 2:** Comparison of diagnostic value of TB detection by mNGS and X-pert MTB/RIF

mNGS	X-pert MTB/RIF		total
	+	-	
+	27	4	31
-	14	3	17
total	41	7	48

*mNGS* metagenomic next-generation sequencing, *TB* tuberculosis

### Value of mNGS for diagnosis and treatment guidance in suspected CAP

Excluding the 50 patients with TB and 4 with NTM infection, we categorized the mNGS results of the remaining 487 patients as pathogen-negative, bacteria-positive (including 1 and ≥ 2 bacteria), fungi-positive (including 1 and ≥ 2 fungi), or bacterial-fungal coinfection.

**Fig. 4:**
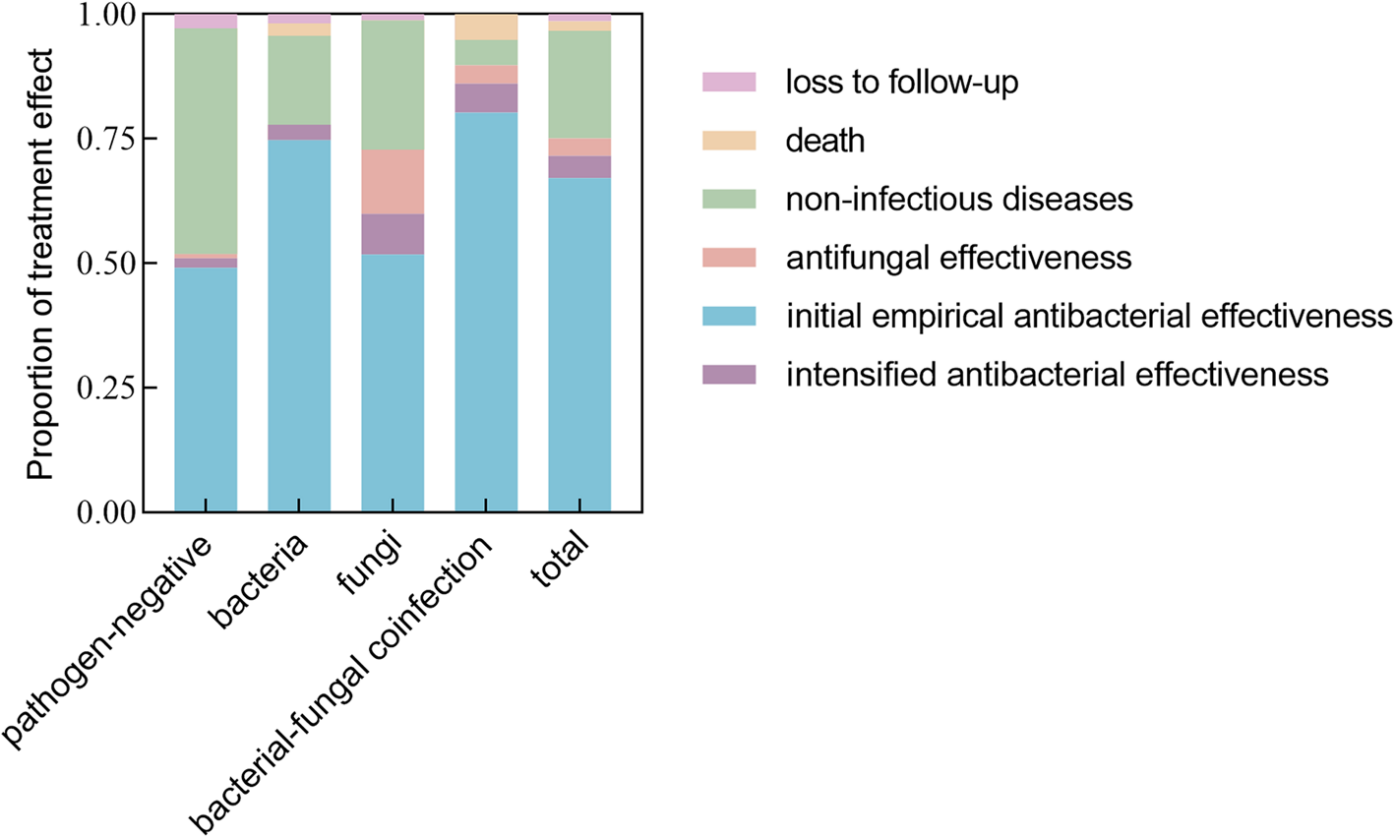
Proportion of treatment effect in different groups of mNGS results

Of the 487 patients included in the evaluation of treatment effect, 67.1% (*N* = 327) improved after initial empirical antibacterial therapy. Of the 106 pathogen-negative cases, 49.1% (*N* = 52) improved with initial empirical antibacterial therapy and 45.3% (*N* = 48) were eventually considered non-infectious diseases, resulting in a negative predictive value of 45.3%. Of the 381 pathogen-positive cases, 72.2% (*N* = 275) improved with initial empirical antibacterial therapy, 81.6% (*N* = 311) after anti-infective therapy, and 15.0% (*N* = 57) were ultimately considered non-infectious diseases, resulting in a positive predictive value of 81.6%. Of the 163 bacteria-positive cases, 77.9% (*N* = 127) improved after antibacterial therapy. Of the 85 fungi-positive cases, 60.0% (*N* = 51) improved after antibacterial therapy and 12.9% (*N* = 11) after antifungal therapy. Of the 137 bacterial-fungal coinfection cases, 86.1% (*N* = 118) improved after antibacterial therapy (Fig. 4).

**Fig. 5:**
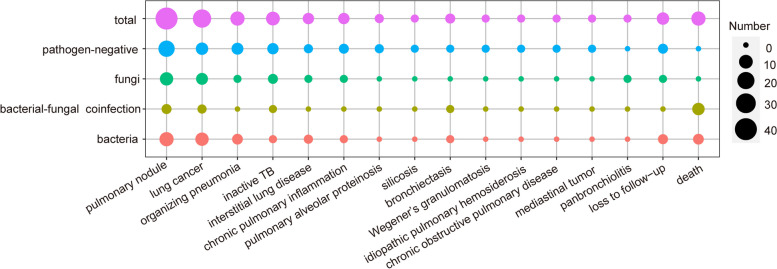
Composition of non-infectious diseases in different groups of mNGS results. The size of the bubble area corresponds to the number of cases of non-infectious diseases. TB, tuberculosis

Of the 106 patients who were pathogen-negative, 52 (49.1%) improved after initial empirical antibacterial treatment, 2 with intensified antibacterial treatment, and 1 with antifungal treatment. The remaining 51 cases did not show significant improvement. 48 of these were ultimately diagnosed as non-infectious diseases (Figs. 1 and 4) after relevant tests (including pathology) or 1 year of follow-up, and 3 were lost to follow-up. These non-infectious cases included 17 cases of pulmonary nodules, 7 lung cancers, 6 organizing pneumonia, 5 inactive TB, 3 chronic pulmonary inflammation, 2 interstitial lung diseases, 2 pulmonary alveolar proteinosis, 1 silicosis, 1 bronchiectasis, 1 Wegener’s granulomatosis, 1 idiopathic pulmonary hemosiderosis, 1 chronic obstructive pulmonary disease, and 1 mediastinal tumor (Fig. 5).

Bacteria were detected by mNGS in 163 patients; of these, 122 (74.8%) improved after initial empirical antibacterial treatment, and 5 improved with intensified antibacterial treatment. Of the 36 cases that did not show significant improvement, 4 died during hospitalization, and 29 were eventually diagnosed as non-infectious diseases (Figs. 1 and 4) after relevant tests or 1 year of follow-up and 3 cases were lost to follow-up. These non-infectious cases included 11 cases of pulmonary nodules, 9 lung cancers, 4 organizing pneumonia, 2 interstitial lung diseases, 1 inactive TB, 1 chronic pulmonary inflammation, and 1 bronchiectasis (Fig. 5).

Fungi were detected by mNGS in 85 patients; of these, 44 (51.8%) improved after initial empirical antibacterial treatment, 7 after intensified antibacterial treatment, and 11 after subsequent adjustment to antifungal therapy (including 5 out of 40 molds, 6 out of 40 yeasts and 0 out of 5 mold-yeast coinfections). The remaining 23 cases did not show significant improvement. Of these, 22 cases were diagnosed as non-infectious diseases (Figs. 1 and 4) after relevant tests or 1 year of follow-up, including 9 cases of pulmonary nodules, 6 lung cancers, 1 organizing pneumonia, 1 interstitial lung disease, 3 inactive TB, 1 chronic pulmonary inflammation, and 1 panbronchiolitis (Fig. 5), and 1 was lost to follow up.

Bacterial-fungal coinfection was detected by mNGS in 137 patients; 110 (80.3%) improved after initial empirical antibacterial treatment, 8 after intensive antibacterial treatment, and 5 after subsequent adjustment to antifungal therapy (including 1 out of 50 molds, 11 out of 71 yeasts and 3 out of 16 mold-yeast coinfections). The remaining 14 cases did not show significant improvement. Of these, 7 had died during hospitalization, and 7 were eventually diagnosed with non-infectious diseases (Figs. 1 and 4) after relevant tests or 1 year of follow-up. These included 3 cases of pulmonary nodules, 2 lung cancers, 1 inactive TB, and 1 bronchiectasis (Fig. 5).

## Discussion

In this study, we analyzed the mNGS test results of 555 BALF samples to investigate its diagnostic performance and clinical value in terms of treatment guidance for patients with suspected CAP. We found that mNGS had advantages for the detection of certain pathogens, although most patients responded to empirical antibiotics; therefore, mNGS was not considered to be an essential routine test.

mNGS cannot be used as the gold standard for the etiological diagnosis of CAP and should be supplemented with other microbiological tests to maximize its effectiveness. Of the 516 samples that were sent for both mNGS and sputum culture, the positivity rate of mNGS was significantly higher than that of conventional microbial culture, which is consistent with the findings of Liang et al. [22]. Conventional microbiological cultures may have a low positivity rate because of false-negative results due to empirical antibiotic treatment prior to the collection of sputum samples and finicky culture conditions for certain pathogenic bacteria [15]. In contrast, the mNGS assay has been shown to be less affected by prior antibiotic exposure than microbial culturing [11, 26]. However, the high positivity rate of mNGS may be explained by false-positive results, due to the following reasons: (1) a failure to manage contaminating microbes, which are ubiquitous and may include normal human flora or may be present in reagents, labware, or the environment [6, 13, 25]; and (2) the respiratory tract is characterized by the presence of numerous normal oral flora, commensal organisms, and colonizers [7], which may be mixed in BALF and difficult to differentiate from pathogens [14, 16]. Furthermore, in cases of double-positive results by mNGS and sputum culture, the pathogens detected by the two were not identical; in some cases, mNGS detected more pathogens than the sputum culture, and in others, the pathogens detected by the two methods were completely different. Consequently, mNGS results should be analyzed objectively. If two methods of detection are consistent, the results are more reliable; if they are not identical, a comprehensive evaluation is recommended.

Based on a patient’s clinical symptoms, laboratory tests, and chest imaging, if TB is suspected, the X-pert MTB/RIF examination should be preferred over mNGS. Using mNGS technology, we detected 33 cases of MTB and 4 cases of NTM; by comparing mNGS and X-pert MTB/RIF, we found that the diagnostic sensitivity of mNGS for TB was not superior to that of X-pert MTB/RIF. These findings are consistent with those of Chen P. et al. [4] and Zhou et al. [35]. In contrast, Shi et al. [27] found that the sensitivity of mNGS for the diagnosis of TB in BALF samples was comparable with that of X-pert MTB/RIF, which was not consistent with our results, possibly because of the high rate of false-negative TB diagnoses by mNGS in our study. These 14 false-negative results may be due to the thicker cell walls of MTB, which are more difficult to rupture to allow the release of sufficient nucleic acid for the mNGS assay [27].

Notably, we detected 6 cases of *Pneumocystis jirovecii* and 2 cases of *Cryptococcus neoformans* using mNGS; all were diagnosed in conjunction with the patients’ other test results and diagnostic treatment effects, which confirmed the accuracy of mNGS. Therefore, mNGS has a unique diagnostic advantage for certain pathogenic microorganisms, such as MTB, NTM, *C. neoformans*, and *P. jirovecii*. Similarly, many studies [3, 27, 31, 34, 35] have demonstrated that mNGS facilitates the detection of these pathogens.

In our study, 55 patients with pathogen-negative mNGS results improved after anti-infective therapy. This may be attributed to the following reasons: (1) false-negative results due to technical reasons, as mNGS detects nucleic acid fragments, and intracellular bacteria, fungi with cell walls, and TB are more resistant to breakage for the extraction of nucleic acid, resulting in a corresponding decrease in the detection efficacy of mNGS [2, 20, 27]; (2) incomplete databases were used, resulting in inaccurate annotation of the sequences of detected pathogenic microorganisms; (3) bioinformatics analysis errors resulting in under-reporting of disease-causing microorganisms; (4) excessively high levels of human nucleic acids in the specimen; (5) the load of pathogenic microorganisms in the sample was below the lower limit of detection for mNGS, or the amount of sequencing data was too low to identify the pathogenic microorganisms [23] (for example, most patients underwent empirical anti-infective therapy prior to BALF collection, resulting in a significant reduction in the pathogenic microbial load); and (6) irregular sampling (e.g., failure to perform adequate irrigation at the lesion site) [1]. Our findings showed that a reliable negative result helped to exclude infection, although the value of excluding infection was limited. Similar findings were reported previously [12, 14, 26]. Miao et al. [26] found that the diagnostic performance (including negative predictive value) of mNGS exceeded that of commonly used assays; however, its negative predictive value reached only 37.4%, and a negative mNGS result could not completely exclude infection. In the present study, the positive and negative predictive values of mNGS were 81.6% and 45.3%, respectively. Therefore, the value of mNGS positivity in diagnosing infection is greater than the value of its negativity in ruling out infection.

We determined that initial empirical antibacterial therapy was effective in 67.1% of patients with suspected CAP, indicating that the majority of patients with suspected CAP benefit from empirical therapy and mNGS is not necessary as a routine test. In bacteria-positive cases, 77.9% of patients responded to antibacterial therapy, whereas only 12.9% of fungi-positive cases improved with antifungal therapy. Therefore, if fungi are detected, antifungal therapy should not be given indiscriminately. Treatment should not be based on the etiological results of mNGS alone; the patient’s immune status, clinical symptoms, chest imaging, relevant laboratory tests, and the effect of the initial antibacterial therapy should be taken into consideration to develop appropriate treatment strategies.

This study had certain limitations. First, viruses were not included in the pathogens detected by mNGS. Second, the tracking time of lung nodules was insufficient, and all were considered to be inflammatory nodules with or without inert lung cancer, which warrants further investigation.

## Conclusions

In summary, as an emerging pathogenetic assay, mNGS played an important role in the diagnosis of certain pathogens (e.g., MTB, NTM, *C. neoformans*, and *P. jirovecii*). Furthermore, it had the advantage of high sensitivity, and its diagnostic accuracy was enhanced by the synergy of conventional pathogenetic tests. However, most patients with suspected CAP responded to empirical treatment, and mNGS was therefore not necessary as a routine test. In addition, the limitations of mNGS should be recognized, including the limited value of negative results to rule out infection and the fact that its detection of fungi should not be treated with indiscriminate antifungal therapy. Overall, mNGS reports should be interpreted critically. However, future prospective studies with larger sample sizes are required to further explore the etiological diagnostic value of mNGS for patients with suspected CAP.

## Data Availability

Demographic and clinical data of the patients which involved in this study were extracted from the patient’s electronic medical record in Renmin Hospital of Wuhan University. The datasets generated and analyzed during the current study are not publicly available due to privacy or ethical restrictions but are available from the corresponding author on reasonable request.

## Abbreviations

mNGS: metagenomic next-generation sequencing
CAP: Community-acquired pneumonia
TB: Tuberculosis
PCR: Polymerase chain reaction
BALF: Bronchoalveolar lavage fluid
ITS: Internal transcribed spacer
NCBI: National Center for Biotechnology Information
MTB: *Mycobacterium tuberculosis*
NTM: Non-tuberculous mycobacteria
